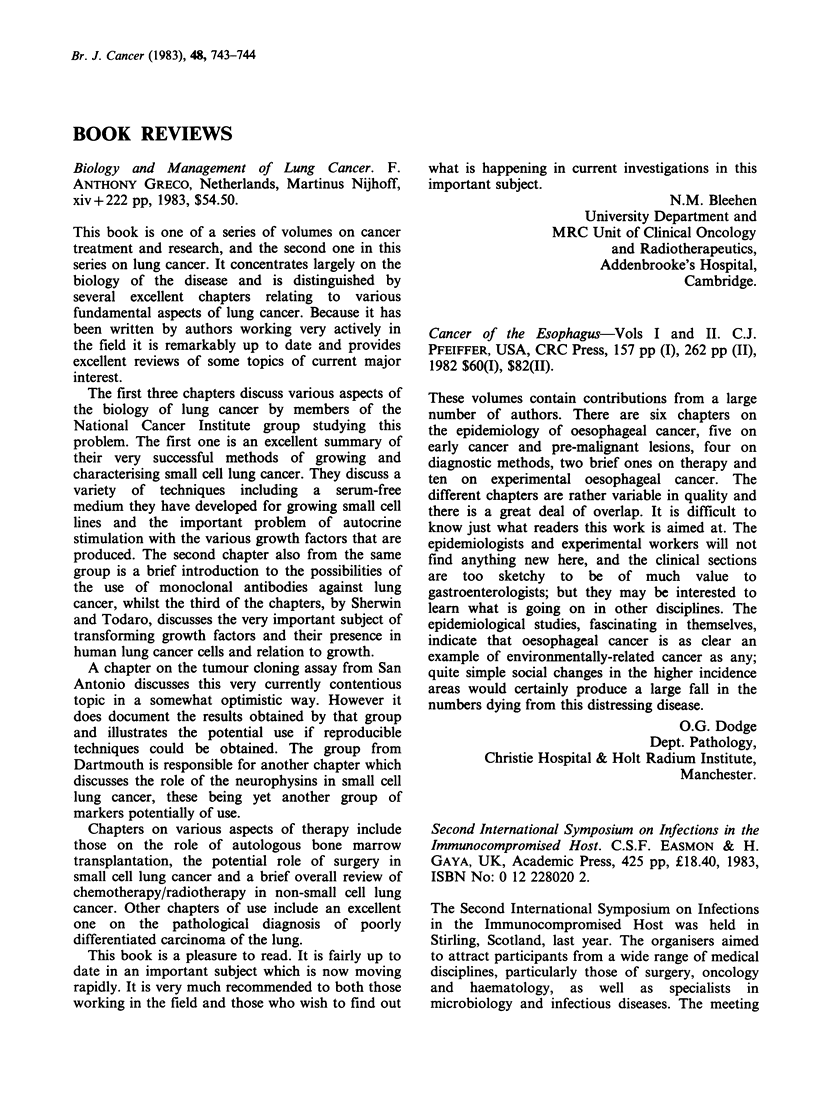# Cancer of the Esophagus—Vols I and II

**Published:** 1983-11

**Authors:** O.G. Dodge


*Cancer of the Esophagus*—Vols I and II. C.J. PFEIFFER, USA, CRC Press, 157 pp (I), 262 pp (II), 1982 $60(I), $82(II).

These volumes contain contributions from a large number of authors. There are six chapters on the epidemiology of oesophageal cancer, five on early cancer and pre-malignant lesions, four on diagnostic methods, two brief ones on therapy and ten on experimental oesophageal cancer. The different chapters are rather variable in quality and there is a great deal of overlap. It is difficult to know just what readers this work is aimed at. The epidemiologists and experimental workers will not find anything new here, and the clinical sections are too sketchy to be of much value to gastroenterologists; but they may be interested to learn what is going on in other disciplines. The epidemiological studies, fascinating in themselves, indicate that oesophageal cancer is as clear an example of environmentally-related cancer as any; quite simple social changes in the higher incidence areas would certainly produce a large fall in the numbers dying from this distressing disease.

O.G. Dodge
Dept. Pathology,
Christie Hospital & Holt Radium Institute,
Manchester.